# Supplementary material for: Scanning tunnelling spectroscopy as a probe of multi-Q magnetic states of itinerant magnets
Source: Nat Commun. 2017 Feb 8;8:14317. doi: 10.1038/ncomms14317 (PMC5309833; doi:10.1038/ncomms14317)
Supplement: Supplementary Information — Supplementary Figures 1-2, Supplementary Notes 1-2 and Supplementary References [file ncomms14317-s1.pdf]

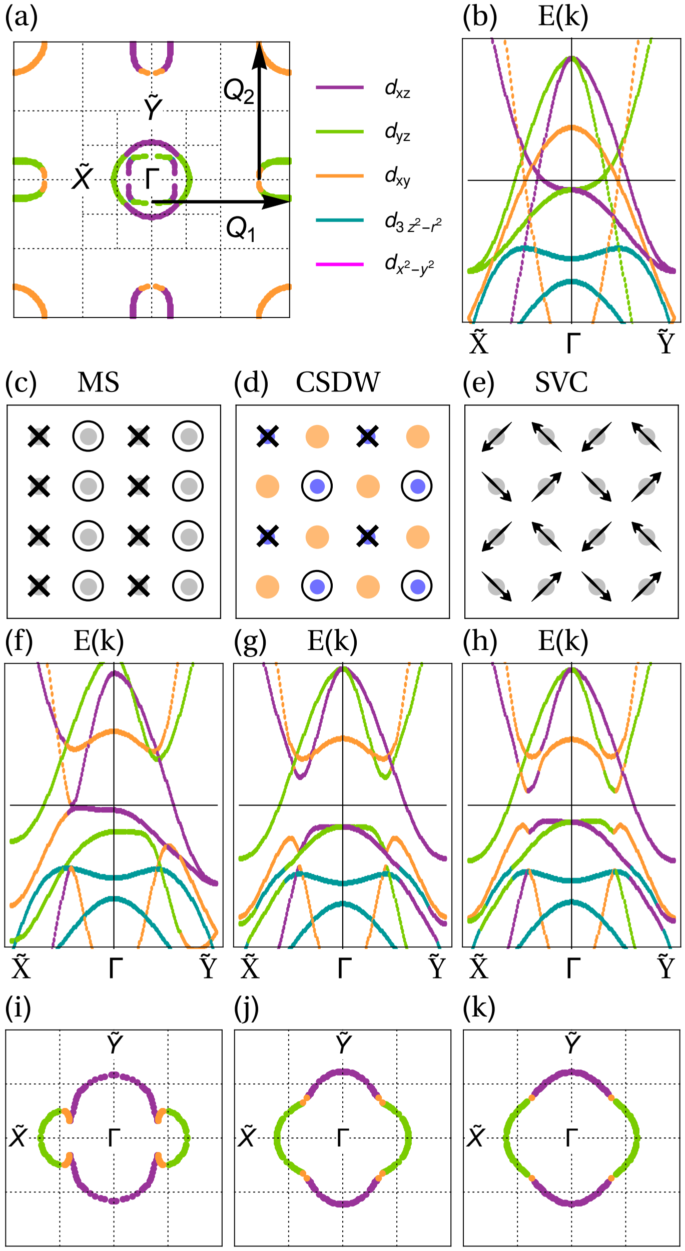

Supplementary Figure 1. **Band reconstructions of magnetic states.** (a) Fermi surface of the normal state and (b) its band dispersion $E(k)$ along high symmetry lines in the folded BZ (FBZ) obtained by folding the original BZ by $\mathbf{Q_1}$ and $\mathbf{Q_2}$. The colors represent the main orbital content, specified in the legend. (c-e) The three SDW states MS (c), CSDW (d) with out-of-plane oriented moments and (π, π) charge order with orange (blue) indicating high (low) electron density n, and SVC (e). The band dispersions (f)-(h) and Fermi surfaces in the FBZ (i)-(k) for the three respective SDW phases in (c-e).

SUPPLEMENTARY NOTE 1

For the band structure relevant to the iron pnictides, three hole-like and two electron-like bands cross the Fermi level, with two degenerate nesting vectors $\mathbf{Q_1} = (\pi, 0)$ and $\mathbf{Q_2} = (0, \pi)$, as shown in Supplementary Figure 1(a,b). The corresponding RPA spin susceptibility is peaked at $\mathbf{Q_1}$ and $\mathbf{Q_2}$, and in general the spin ordered state will be a combination of both ordering vectors, $\mathbf{M}(\mathbf{r}) = \sum_{l=1,2} \mathbf{M}_l \exp(i\mathbf{Q}_l \cdot \mathbf{r})$.[1–3] The spin structures of the three distinct magnetic states are shown in Supplementary Figures 1(c)-(e). For these systems, the prevalent magnetic phase is the MS state with in-plane moments along the antiferromagnetic ordering vector. Interestingly, the new experimentally

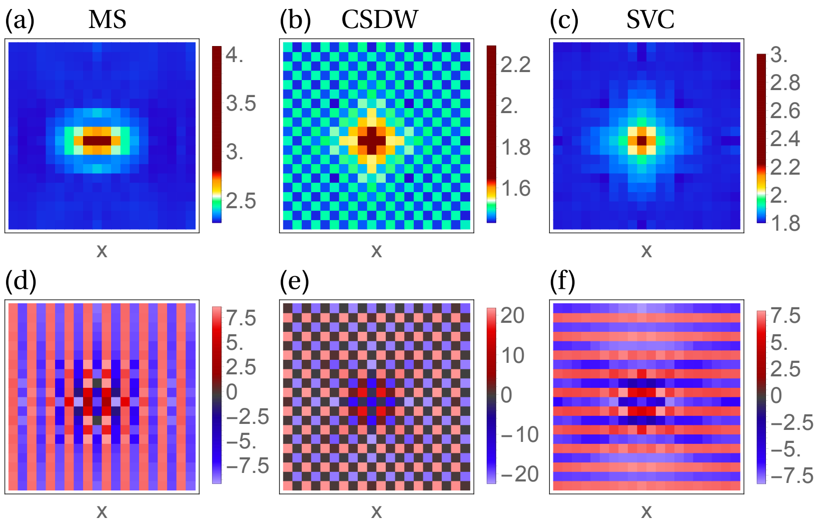

Supplementary Figure 2. **Spin-summed and spin-resolved spectroscopy around a nonmagnetic impurity.** Total (spin-summed) LDOS $N(\mathbf{i},\omega)$ at $\omega = 0$ around a $V_p = 0.5$ eV non-magnetic impurity in the (a) MS, (b) CSDW, and (c) SVC states. (d)-(f) Local spin polarization percent $\mathbf{P}(\mathbf{i}) \cdot \hat{\mathbf{l}}$ [%] of the corresponding cases in the upper row with $\hat{\mathbf{l}} = \hat{\mathbf{z}}, \hat{\mathbf{z}}$ and $\hat{\mathbf{x}}$, respectively.

observed magnetic phase exhibits a spin reorientation from in-plane to out-of-plane, with moments along the c axis.[4] The relevant spin structures for this new phase are thus collinear single-$\mathbf{Q}$ MS and double-$\mathbf{Q}$ CSDW states with out-of-plane moments, which we consider in this work (Supplementary Figures 1(c)-(d)).

In order to compare the resulting electronic properties of the three SDW states at an equal footing, we fix the temperature to $\kappa_B T = 10$ meV, the electron filling $n = 5.88$, and the interaction parameters $U = 0.95$ eV. In this region of parameter space, the CSDW state is the global minimum.[5] The other two magnetic states are local minima, which may be stabilized self-consistently by applying restrictions to the fields. In this way all three magnetic states are generated from the same normal state [Supplementary Figures 1(a)-(b)]. Supplementary Figures 1(f)-(k) show the reconstructed Fermi surfaces and band structures of the three different SDW states along high symmetry lines in the folded Brillouin zone (FBZ) $(-\pi/2 < k_x, k_y < \pi/2)$. In the single-$\mathbf{Q}$ MS state only SDW gaps at momenta connected by the ordering vector $\mathbf{Q_1}$ open, leaving the direction parallel to the stripes metallic, and thus resulting in a C_2 symmetric band dispersion and Fermi surface shown in Supplementary Figures 1(f) and 1(i), respectively. In the double-$\mathbf{Q}$ states, the gaps open at momenta connected by both $\mathbf{Q_1}$ and $\mathbf{Q_2}$, resulting in two very similar band reconstructions with almost identical Fermi surfaces, as seen by comparison of Supplementary Figures 1(g)-(h) and 1(j)-(k), despite their very different magnetic structures in real space.

SUPPLEMENTARY NOTE 2

In this section we briefly analyze the effects of a non-magnetic impurity introduced by the term

$$\mathcal{H}_p = V_p \sum_{\mu\sigma} c^{\dagger}_{\mathbf{i}^*\mu\sigma} c_{\mathbf{i}^*\mu\sigma}, \tag{1}$$

which adds a local spin-less potential at site $\mathbf{i}^*$. The orbitally diagonal potential V_p is a good approximation in these systems[6]. We calculate the projected spin resolved LDOS

$$N_{\sigma\sigma'}(\mathbf{i},\omega) = -\frac{1}{\pi}\mathrm{Im}(\mathcal{G}_{\sigma\sigma'}(\mathbf{i},\omega)) = -\frac{1}{\pi}\mathrm{Im}\sum_{n,\mu}\frac{u^n_{\mathbf{i}\mu\sigma}u^n_{\mathbf{i}\mu\sigma'}}{\omega - E_n + i\eta}, \tag{2}$$

to get the total LDOS $N(\mathbf{i},\omega) = \sum_{\sigma\sigma'} N_{\sigma\sigma'}(\mathbf{i},\omega)$ at energy ω, and the local spin-polarization of the electrons at the Fermi level ($\omega = 0$), $\mathbf{P}(\mathbf{i}) = \mathrm{Tr}\left(\boldsymbol{\sigma}_{\sigma\sigma'} N_{\sigma\sigma'}(\mathbf{i},0)\right)/N(\mathbf{i},0))$.

Supplementary Figures 2(a)-(c) display the resulting LDOS at $\omega = 0$ around a $V_p = 0.5$ eV potential placed in each of the three different magnetic states. In the MS state [Supplementary Figure 2(a)] the impurity reflects the broken C_4 symmetry of the homogeneous system. By contrast, the tetragonal symmetry is preserved around the potential in both double-**Q** phases as seen from Supplementary Figures 2(b) and 2(c). In the CSDW case there is a (π, π) modulation in $N(\mathbf{i}, \omega)$, arising from the charge order at $\mathbf{q} = \mathbf{Q_1} + \mathbf{Q_2}$ of the homogeneous state. The (π, π) charge modulation constitutes a potentially strong STM fingerprint of the CSDW ordered state. The amplitude of this modulation, however, may be too small to be easily detected by tunneling spectroscopy. Finally, nonmagnetic impurities couple directly to the charge density, and thus potentials placed in any site of a system with homogeneous charge density will show identical $N(\mathbf{i}, \omega)$ features. That is, a given potential V_p will show the same C_2 symmetric LDOS at any site in the MS state, and the same C_4 symmetric LDOS at any site in the SVC state. In the case of the CSDW state, on the other hand, there are two inequivalent charge density sites arising from the associated (π, π) CDW, and therefore this state will show two different LDOS patterns around potentials. This will in principle allow one to distinguish the SVC and CSDW phases, but the distinction between the two LDOS features relies on the detection of amplitude differences, since tetragonal symmetry is still preserved at all sites.

The local spin polarization $\mathbf{P}(\mathbf{i})$ gives complementary information that would, in principle, allow one to distinguish between the CSDW and the SVC phases from the spin-polarized tunneling conductance. We show in Supplementary Figures 2(d)-(f) the polarization at the Fermi level for the relevant spin projection $\hat{\mathbf{l}}$ of the three magnetic states ($\mathbf{P}(\mathbf{i}) \cdot \hat{\mathbf{l}} \propto \mathbf{M_i} \cdot \hat{\mathbf{l}}$). This property is related to the magnetic contrast measured in a spin-polarized STM experiment[7]. The polarization of the single-**Q** state in Supplementary Figure 2(d) consists of $\mathbf{Q_1}$ modulated stripes for the $\hat{\mathbf{l}} = \hat{\mathbf{z}}$ projection. The $\hat{\mathbf{l}} = \hat{\mathbf{x}}$ and $\hat{\mathbf{l}} = \hat{\mathbf{y}}$ components have no polarization, since $\mathbf{M_i} \cdot \hat{\mathbf{l}} = 0$ in the xy plane [Supplementary Figure 1(c)]. In the case of the CSDW state, the relevant projection is also the $\hat{\mathbf{z}}$ axis [Supplementary Figure 1(d)], where half of the sites appear with alternating polarization and the other half are not polarized, resulting from an equal superposition of $\mathbf{Q_1}$ and $\mathbf{Q_2}$ parallel spin density waves. Note that the C_4 symmetry is preserved in the polarization pattern around the impurity in this state. The last magnetic state, the SVC, with a coplanar spin structure [Supplementary Figure 1(e)] has two relevant spin projections, the in-plane $\hat{\mathbf{l}} = \hat{\mathbf{x}}$ and $\hat{\mathbf{l}} = \hat{\mathbf{y}}$. $\mathbf{Q_2}$ modulated stripes can be seen in Supplementary Figure 2(f) for $\hat{\mathbf{l}} = \hat{\mathbf{x}}$, with a local C_2 symmetric polarization pattern around the impurity. The perpendicular $\hat{\mathbf{l}} = \hat{\mathbf{y}}$ polarization (not shown) consists of $\mathbf{Q_1}$ stripes, similar to those in the MS state [Supplementary Figure 2(d)]. The two double-**Q** phases are now clearly discernible, with distinctive local and global spin-polarization tunneling.

SUPPLEMENTARY REFERENCES

[1] I. Eremin and A. V. Chubukov, Magnetic degeneracy and hidden metallicity of the spin-density-wave state in ferropnictides, *Phys. Rev. B* **81**, 024511 (2010).

[2] J. Lorenzana, G. Seibold, C. Ortiz, and M. Grilli, Competing Orders in FeAs Layers, *Phys. Rev. Lett.* **101**, 186402 (2008).

[3] G. Giovannetti, C. Ortix, M. Marsman, M. Capone, J. van den Brink, and J. Lorenzana, Proximity of iron pnictide superconductors to a quantum tricritical point, *Nature Commun.* **2**, 398 (2011).

[4] F. Waßer *et al.*, Spin reorientation in $Ba_{0.65}Na_{0.35}Fe_2As_2$ studied by single-crystal neutron diffraction, *Phys. Rev. B* **91**, 060505(R) (2015).

[5] M. N. Gastiasoro and B. M. Andersen, Competing magnetic double-Q phases and superconductivity-induced reentrance of C_2 magnetic stripe order in iron pnictides, *Phys. Rev. B* **92**, 140506(R) (2015).

[6] K. Nakamura, R. Arita, and H. Ikeda, First-principles calculation of transition-metal impurities in LaFeAsO, *Phys. Rev. B* **83**, 144512 (2011).

[7] R. Wiesendanger, Spin mapping at the nanoscale and atomic scale, *Rev. Mod. Phys.* **81**, 1495 (2009).
